# Integrative multi-omics analysis reveals the interaction mechanisms between gut microbiota metabolites and ferroptosis in rheumatoid arthritis

**DOI:** 10.3389/fimmu.2025.1608262

**Published:** 2025-07-09

**Authors:** Lifang Liang, Huaguo Liang, Min He, Huiling Zhang, Peifeng Ke

**Affiliations:** ^1^ Department of Laboratory Medicine, The Second Affiliated Hospital of Guangzhou University of Chinese Medicine, Guangzhou, China; ^2^ Department of Laboratory Medicine, Guangdong Provincial Hospital of Chinese Medicine, Guangzhou, China; ^3^ The Second Clinical Medical College, Guangzhou University of Chinese Medicine, Guangzhou, China; ^4^ State Key Laboratory of Oncology in South China, Guangdong Key Laboratory of Nasopharyngeal Carcinoma Diagnosis and Therapy, Guangdong Provincial Clinical Research Center for Cancer, Sun Yat-sen University Cancer Center, Guangzhou, China

**Keywords:** rheumatoid arthritis, ferroptosis, gut microbiota, GPX3, MYC, gutMGene database

## Abstract

**Background:**

Rheumatoid arthritis (RA) is an autoimmune disease characterized by chronic synovitis and joint destruction. To systematically investigate the regulatory relationship between key ferroptosis genes and gut metabolites in RA, this study employed an integrative multi-omics approach combined with machine learning algorithms and single-cell transcriptomic data, identifying and validating GPX3 and MYC as potential critical ferroptosis regulators in RA.

**Methods and results:**

First, 16 candidate genes were obtained by intersecting WGCNA, differential expression analysis results, and targets related to ferroptosis and gut microbiota. Following cross-validation with machine learning approaches including LASSO, SVM, and RFE-RF, GPX3 and MYC were ultimately identified as crucial genes. GSVA and GSEA analyses revealed that high expression of GPX3 and MYC was enriched in interferon response and TNFA signaling pathways, while their low expression was associated with fatty acid metabolism and oxidative phosphorylation pathways. Further single-cell RNA sequencing analysis demonstrated that MYC was expressed in multiple immune cell types, particularly in CD4+ T cells and NK cells. Ferroptosis scoring for CD8+ T cells and subsequent cell communication analysis revealed stronger interactions between CD8+ T cells with higher ferroptosis scores and other immune cells through IFN-II and CCL signaling, further intensifying the activation of the inflammatory microenvironment. Additionally, molecular docking analysis of GPX3 and MYC with the gut metabolites Diosgenin and Differentiation-inducing factor 3 (DIF-3) respectively showed that the GPX3-Diosgenin complex had the lowest binding energy, and a 100 ns molecular dynamics simulation was performed on this complex. Results showed good stability of the complex across indicators such as RMSD, RMSF, SASA, and radius of gyration, suggesting that Diosgenin may intervene in ferroptosis and inflammatory injury in RA by binding to and modulating GPX3 function.

**Conclusion:**

This study elucidated the multifaceted mechanisms of GPX3 and MYC in RA pathogenesis and preliminarily validated the potential role of gut metabolites in mediating ferroptosis regulation, offering novel theoretical foundations and potential strategies for diagnostic biomarker screening and targeted therapy in RA.

## 1 Introduction

Rheumatoid arthritis (RA) is a chronic, systemic autoimmune disease characterized primarily by chronic inflammation and progressive destruction of the joint synovium, often resulting in joint deformity and functional disability, significantly impairing patients’ quality of life (1, 2). The pathogenesis of RA is believed to involve immune system abnormalities resulting from interactions between environmental factors (such as smoking and infection) and genetic susceptibility (3). Immune system dysfunction results in the production of autoantibodies and excessive release of inflammatory mediators, ultimately causing joint damage (4). Despite significant advances in RA diagnosis and treatment in recent years, such as the application of biologics and targeted synthetic disease-modifying antirheumatic drugs, a subset of patients still exhibits poor response to current treatments (1).

In recent years, the roles of gut microbiota and ferroptosis in RA have gradually attracted attention. Gut microbiota interacts with the host immune system through their metabolites, contributing to the progression of RA (5). Studies have shown significant dysbiosis in the gut microbiota of RA patients, characterized by an increase in Lactobacillus and Streptococcus, and a decrease in Bacteroides and Faecalibacterium (6). Short-chain fatty acids derived from gut microbial metabolism regulate the differentiation and function of regulatory T cells, thereby influencing immune tolerance and inflammatory responses (7). Ferroptosis, a form of iron-dependent cell death, plays a critical role in various pathological processes, including neurodegenerative diseases, cancer, and immune diseases (8). Ferroptosis not only promotes oxidative stress but also exacerbates joint inflammation and injury by inducing lipid peroxidation damage in cartilage and synovial cells within joint tissues (9). Additionally, ferroptosis may influence immune responses in RA by modulating immune cell functions, including the activation and differentiation of T cells and macrophages, during iron metabolism and oxidative stress (10).

Recent studies have confirmed a complex and close interrelationship among gut microbiota, ferroptosis, and RA. Wang et al. (11) found that modulating gut microbiota and short-chain fatty acid metabolism could inhibit ferroptosis induced by lipid oxidative stress in synovial tissues, thereby preventing RA-associated damage. Furthermore, ferroptosis can exacerbate RA-associated inflammation and joint injury by upregulating the expression of peptidylarginine deiminase 4 (PAD4), thus influencing gut dysbiosis and metabolic disturbances (12, 13). Thus, integrating multi-omics data to explore the potential associations among gut microbiota, ferroptosis, and RA could help elucidate RA’s complex pathogenesis and identify novel targets for diagnosis and treatment.

**Figure 1:**
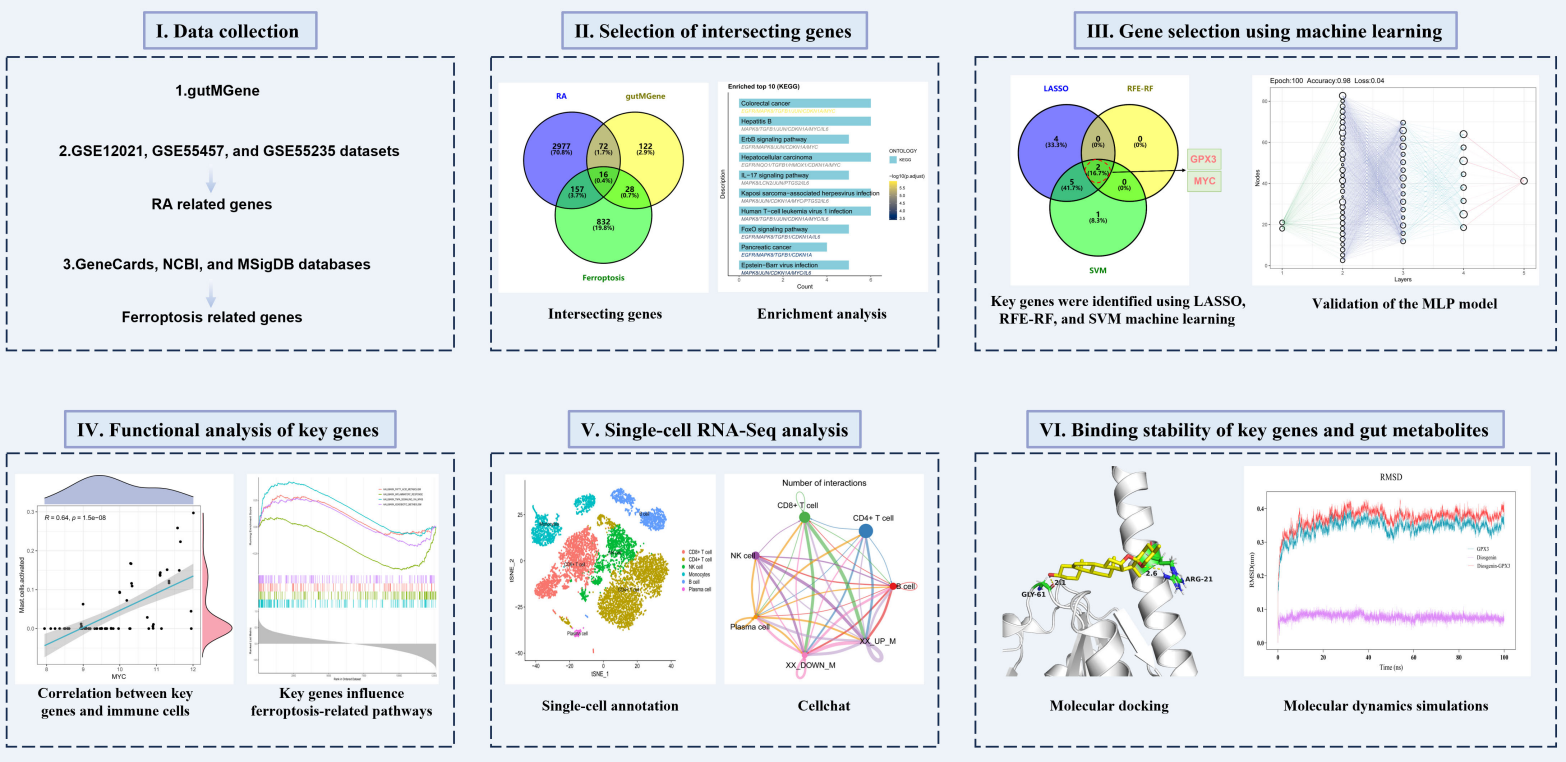
Schematic diagram of the research workflow and analytical strategy.

This study aimed to deeply investigate the interrelationships among gut microbiota, ferroptosis, and RA through integrated multi-omics analysis combined with network pharmacology and machine learning approaches. We obtained gut microbiota-related genes from the gutMGene database and screened ferroptosis-related genes from GeneCards, NCBI, and MSigDB databases. Subsequently, RA-associated genes were identified by analyzing RA-related datasets, including GSE12021, GSE55457, and GSE55235, and intersection analysis was employed to uncover potential associations among gut microbiota, ferroptosis, and RA. Key genes were screened using machine learning methods such as Least Absolute Shrinkage and Selection Operator (LASSO), recursive feature elimination-random forest (RFE-RF), and support vector machine (SVM), and further validated using a multilayer perceptron (MLP) model. To further elucidate the regulatory mechanisms of the key genes, molecular docking and molecular dynamics simulations were performed on gut metabolites influencing these genes, assessing their binding affinity and stability. The schematic diagram of the research workflow and analytical strategy is shown in **Figure 1**. Through these multidimensional analyses, this study aims to provide novel theoretical insights into the interactions among gut microbiota, ferroptosis, and RA.

## 2 Materials and methods

### 2.1 Data collection of gut microbiota metabolites and ferroptosis-related genes

The gutMGene database is a comprehensive resource providing extensive information on gut microbial metabolites and their interactions with human genes (14). In this study, gut microbiota-associated metabolites and their corresponding human targets were systematically collected from the gutMGene database (http://bio-computing.hrbmu.edu.cn/gutmgene) to establish a metabolite-target association dataset. Additionally, to screen ferroptosis-related genes, we searched the GeneCards (https://www.genecards.org/), NCBI Gene (https://www.ncbi.nlm.nih.gov/gene/), and MSigDB (https://www.gsea-msigdb.org/gsea/msigdb/) databases using “Ferroptosis” as a keyword, obtaining a deduplicated candidate gene set. The authenticity of this article has been validated by uploading the key raw data onto the Research Data Deposit public platform (www.researchdata.org.cn), with the approval RDD number as RDDB2025916508.

### 2.2 Screening and identification of RA-associated genes

In this study, three RA-related synovial tissue expression datasets—GSE55457, GSE55235, and GSE12021—were selected from the GEO database (https://www.ncbi.nlm.nih.gov/geo/). To guarantee data consistency and comparability, all datasets were derived from the same chip platform (Affymetrix Human Genome U133A Array, GPL96). The dataset selection was based on the following criteria: (1) Synovial tissue samples must be clearly identified as from RA patients and normal controls; (2) Each dataset must include no fewer than 5 RA and 5 control samples; (3) The datasets must originate from the same microarray platform to ensure probe annotation consistency; (4) Both expression matrices and sample annotation information must be fully available.

To ensure consistency and comparability of expression matrices across different datasets, we first extracted common genes from the three datasets to unify analytical dimensions. Subsequently, we read the raw expression matrices and performed log2 transformation based on their distribution characteristics to eliminate interference from extreme values. Simultaneously, sample names were standardized by applying a consistent naming format and adding dataset-specific prefixes to differentiate their origins. Regarding data normalization, each dataset was individually normalized using the normalizeBetweenArrays function from the limma package, and after adjusting expression distributions, they were horizontally merged according to gene dimension. To control for systematic biases arising from data sources, batch effects were further corrected using the ComBat function from the sva package. Finally, dimensionality reduction visualization was conducted via principal component analysis (PCA) on data before and after correction to assess the efficacy of batch correction and the discriminative power of sample grouping, ensuring the quality of data integration met the requirements for subsequent co-expression analysis.

Subsequently, differential expression analysis was performed on the merged dataset using the Limma R package with the criteria of |logFC| > 1 and adj.P.Val < 0.05 to screen RA-related differentially expressed genes, and a volcano plot was generated to visualize the results. Additionally, a heatmap depicting the top 50 genes with the most significant expression changes was created using the pheatmap R package to visually illustrate gene expression differences between RA patients and normal controls. To further investigate gene modules associated with RA, weighted gene co-expression network analysis (WGCNA) was conducted. By constructing a gene co-expression network, modules significantly correlated with the RA phenotype were identified, and hub genes within these modules were extracted.

### 2.3 Intersection gene screening and functional enrichment analysis

To explore potential associations among gut microbiota, ferroptosis, and RA, Venn diagram analysis was performed on gut microbiota-related genes, ferroptosis-related genes, and RA-related genes to identify intersection genes. To visually demonstrate the expression patterns of intersection genes, bar plots were generated using the ggplot2 package in R, illustrating the differential expression of these genes between RA patients and healthy controls. To further elucidate the biological functions and signaling pathways of intersection genes, Gene Ontology (GO) and Kyoto Encyclopedia of Genes and Genomes (KEGG) enrichment analyses were conducted using the clusterProfiler R package. GO enrichment analysis covered biological processes (BP), cellular components (CC), and molecular functions (MF) to uncover the biological characteristics of these genes, whereas KEGG enrichment analysis identified key metabolic and signaling pathways. Finally, results from GO and KEGG analyses were visualized using bar plots.

### 2.4 Machine learning-based screening of key genes

To further identify key genes with important regulatory roles in RA, three machine learning methods—LASSO regression, RFE-RF, and SVM—were employed for feature gene selection. First, feature selection was performed using LASSO via the glmnet R package, with the optimal λ value used to identify relevant genes. Next, the RFE-RF method was implemented using the caret and shap R packages, with 10-fold cross-validation used to assess model stability and ultimately select important feature genes. Finally, the SVM method was applied for feature selection using the e1071 R package, with 5-fold cross-validation performed to ensure model robustness. SVM constructs a hyperplane in a high-dimensional space to classify gene expression data and identifies genes that most influence classification outcomes. We integrated the results from LASSO, RFE-RF, and SVM, and used a Venn diagram to identify genes commonly selected by all three methods.

### 2.5 Expression characteristics and correlation analysis of key genes

To further validate the diagnostic value and biological significance of the identified key genes in RA, a series of multilevel analyses was conducted. The predictive ability of key genes in distinguishing RA patients from healthy individuals was assessed using receiver operating characteristic (ROC) curves, and the area under the curve (AUC) was calculated. Next, the correlation between key genes was analyzed using Pearson correlation analysis to evaluate gene-gene expression relationships, and scatter plots were generated to visualize the interaction patterns. Additionally, boxplots were created to compare the expression levels of key genes between RA and healthy control groups, highlighting differential expression.

### 2.6 Validation of key genes using the MLP model

To further validate the predictive ability of key genes in RA diagnosis, a MLP neural network model was constructed for classification analysis. MLP is a feed-forward neural network consisting of an input layer, multiple hidden layers, and an output layer, capable of recognizing complex nonlinear feature patterns. First, we extracted the expression values of key genes from the expression matrix across samples and applied Z-score normalization to each gene to eliminate differences in scales among features. According to sample classification labels (RA group vs. normal group), the dataset was randomly split into training and testing sets at a 7:3 ratio, ensuring roughly balanced class distributions in both subsets. During model training, min-max normalization was applied to further enhance convergence speed and stability. The constructed MLP model consists of one input layer, three hidden layers (containing 32, 16, and 8 nodes, respectively), and one output layer. All hidden layers used ReLU activation functions, with a dropout layer (rate = 0.2) added after each to prevent overfitting; the output layer utilized a sigmoid function for binary classification output. The model was iteratively trained using the Adam optimizer, with binary cross-entropy as the loss function, an initial learning rate set at 0.01, batch size of 16, and training epochs set to 100. After training completion, the model’s classification performance was evaluated on the test set by measuring accuracy and loss, and its diagnostic effectiveness was assessed via ROC curves and AUC values. Additionally, a confusion matrix was used to visually present model predictions, and a network structure diagram was created to illustrate layer connections and weight distributions, thus validating the diagnostic potential of key genes from multiple perspectives.

### 2.7 Single-gene Gene Set Enrichment Analysis and Gene Set Variation Analysis analysis of key genes

To explore the potential regulatory mechanisms of key genes in RA, GSEA and GSVA were performed to evaluate the association between key genes and HALLMARK pathways. GSEA was performed using the clusterProfiler R package to conduct enrichment analysis on HALLMARK pathways. Normalized enrichment scores (NES) and adjusted P-values were calculated based on the expression levels of key genes to identify significantly enriched pathways. GSVA was performed using the GSVA R package to calculate enrichment scores for each sample in HALLMARK pathways. Samples were grouped into high- and low-expression groups based on the expression level of the key gene, and differences in pathway activity were assessed to uncover potential molecular mechanisms involving the key genes.

### 2.8 Immune infiltration analysis

To further explore the regulatory roles of key genes in the RA immune microenvironment, CIBERSORT was used in conjunction with the LM22 reference gene set to quantify the infiltration of 22 immune cell types, and immune cell composition was compared between RA and healthy control groups. Furthermore, Pearson correlation analysis was used to evaluate relationships among immune cell types, and a correlation heatmap was generated to reveal dynamic changes and potential regulatory networks in the RA immune microenvironment. In addition, correlation analysis was conducted to assess the associations between key gene expression and immune cell infiltration levels. Correlation scatter plots were generated to explore potential mechanisms by which key genes may contribute to immune dysregulation in RA.

### 2.9 Single-cell transcriptomic analysis

To investigate the expression patterns of key genes across different cell types and their dynamic changes within the RA immune microenvironment, the GSE159117 dataset was downloaded from the GEO database and subjected to systematic single-cell transcriptomic analysis. The data were processed using the Seurat R package, including steps such as quality control, normalization, dimensionality reduction, and clustering. Cell types were annotated based on known immune cell marker genes, including: CD4+ T cells (CD2, CD4, CCR7, CD3D), CD8+ T cells (CD8A, GNLY, GZMB), NK cells (LTB, NKG7), plasma cells (XBP1, CD27), B cells (MS4A1, CD79A), and monocytes (CD14, LYZ, FCGR3A). The FeaturePlot function was used to visualize the expression of key genes across different cell types, providing an intuitive view of their tissue distribution. To explore ferroptosis-related mechanisms, the AddModuleScore function was used in combination with a ferroptosis gene set to calculate a ferroptosis score for each cell. Furthermore, using the CellChat R package, cell-cell communication patterns were analyzed under high and low ferroptosis score states across different cell types.

### 2.10 Molecular docking and molecular dynamics simulation analysis

To investigate the potential role of gut microbiota-derived metabolites in regulating ferroptosis in RA, gut metabolites interacting with key genes were identified from the gutMGene database, and molecular docking and molecular dynamics simulation were employed to evaluate the stability of their interactions. Molecular docking was conducted using AutoDock 4.2.3 by obtaining the 3D structure of the protein encoded by the key gene (in PDB format) and the structure of the candidate gut metabolite (in SDF format) for docking score calculation. he docking process included protein-ligand structure optimization, grid box configuration, and flexible docking parameter adjustment. The conformation with the lowest binding energy was selected as the candidate complex. A lower binding energy indicates a more stable interaction between the protein and the metabolite.

To further evaluate the stability of the molecular docking complex, a 100-nanosecond molecular dynamics simulation was conducted using GROMACS 2022.3 (15). During the simulation, the AMBER99SB force field was used, and the system was solvated with the SPC/E water model. A suitable number of Na^+^ ions were added to neutralize the total charge of the simulation system. Simulation parameters included: Energy minimization using the steepest descent method; NVT (constant volume and temperature) and NPT (constant pressure and temperature) equilibrations, each for 100 ps at 300K; Production run: 100 ns with a 2 fs time step, recording the trajectory of protein–ligand interactions. The stability and binding capacity of the protein–ligand complex were evaluated by calculating root-mean-square deviation (RMSD), root-mean-square fluctuation (RMSF), number of hydrogen bonds (H-bonds), solvent-accessible surface area (SASA), radius of gyration (Gyrate), and binding free energy (MM/GBSA).

## 3 Results

### 3.1 Collection and screening of target genes

In this study, a total of 276 gut microbiota-derived metabolites and 238 corresponding human gut targets were obtained from the gutMGene database to construct a metabolite–target interaction network. Additionally, 1033 ferroptosis-related genes were identified from GeneCards, NCBI, and MSigDB databases (**Supplementary Table 1**) as a crucial gene set for intersection analysis between RA-related genes and gut microbiota metabolic pathways. To ensure consistency and comparability of integrated expression data from different GEO datasets, we performed rigorous data cleaning and normalization on the three expression datasets: GSE55457, GSE55235, and GSE12021. PCA was employed to visualize samples to validate the effectiveness of normalization and batch correction. **Supplementary Figures 1A, B** illustrate PCA distributions grouped by dataset and disease status before batch effect correction, respectively, clearly showing significant separation among samples based on dataset origin, indicating a strong batch effect. After correction using the ComBat method (**Supplementary Figures 1C, D**), dataset differences among samples significantly decreased, and the clustering structure of RA and normal control groups in principal component space became clearer, indicating that the data processing workflow effectively enhanced data quality and the reliability of subsequent analyses.

**Figure 2:**
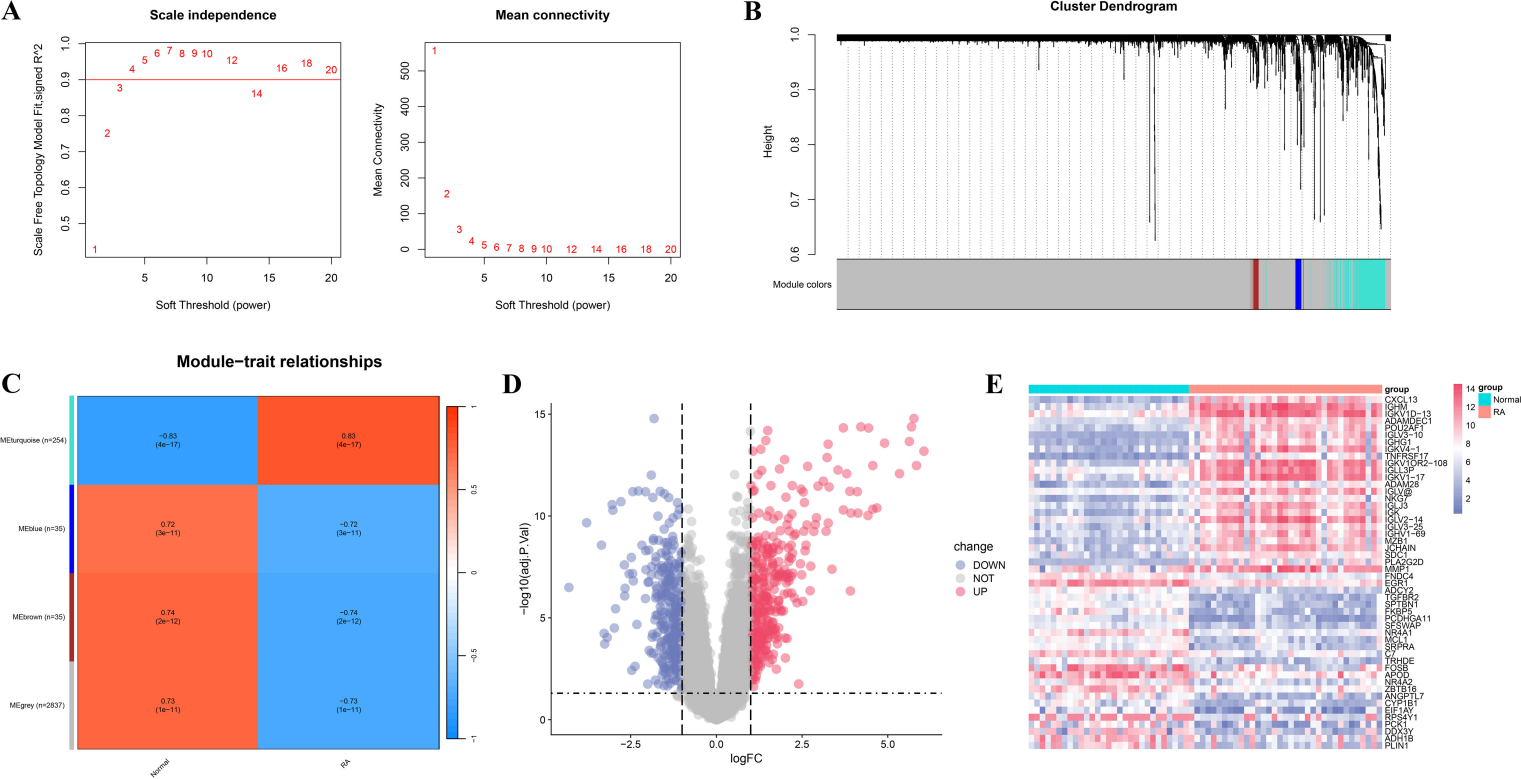
WGCNA and differential expression analysis of RA-related genes. **(A)** Soft-threshold selection plot. The left panel shows the scale independence of the network topology; the right panel illustrates the average connectivity under different soft-thresholding powers. **(B)** Dendrogram and module identification from WGCNA. Different colors represent distinct co-expression gene modules. **(C)** Heatmap showing the correlation between gene modules and clinical phenotypes (RA vs. normal control groups). **(D)** Volcano plot of RA-related differentially expressed genes. Red indicates significantly upregulated genes, blue indicates significantly downregulated genes, and gray denotes non-significant genes. **(E)** Heatmap of significantly differentially expressed genes.

To further identify RA-associated genes, we conducted WGCNA and differentially expressed gene analysis. **Figure 2A** shows the selection of the soft-thresholding power (β) in network topology analysis, used to construct a scale-free network. **Figures 2B, C** shows the gene co-expression network divided into four modules, with 254, 36, 36, and 2837 genes in the turquoise, blue, brown, and gray modules, respectively. Genes in the blue module were enriched in various inflammatory response-related biological processes, such as response to lipopolysaccharide and cytokine activity (**Supplementary Figure 2A**), and KEGG analysis revealed significant enrichment in classical RA-related immune-inflammatory pathways such as IL-17 signaling pathway, TNF signaling pathway, and NF-kappaB signaling pathway (**Supplementary Figure 2B**), suggesting that this module may participate in the regulation of pro-inflammatory signaling in RA. Genes in the turquoise module were broadly enriched in processes such as immune cell activation, lymphocyte differentiation, and immunological synapse formation (**Supplementary Figure 2C**), and KEGG analysis demonstrated significant enrichment in immune regulatory pathways closely related to RA pathogenesis, including Th17 cell differentiation, cytokine–cytokine receptor interaction, and chemokine signaling pathway (**Supplementary Figure 2D**). Modules with a phenotype correlation coefficient greater than 0.5 and a P-value less than 0.05 were selected for subsequent analyses. Specifically, genes from the turquoise, blue, brown, and gray modules were selected for further investigation. Differential expression analysis identified 698 differentially expressed genes, including 382 upregulated and 312 downregulated genes (**Figures 2D, E**). Finally, the union of WGCNA module genes and differentially expressed genes was taken and duplicates removed, yielding 3,222 candidate RA-related genes.

### 3.2 Screening of intersecting genes and functional enrichment analysis

**Figure 3:**
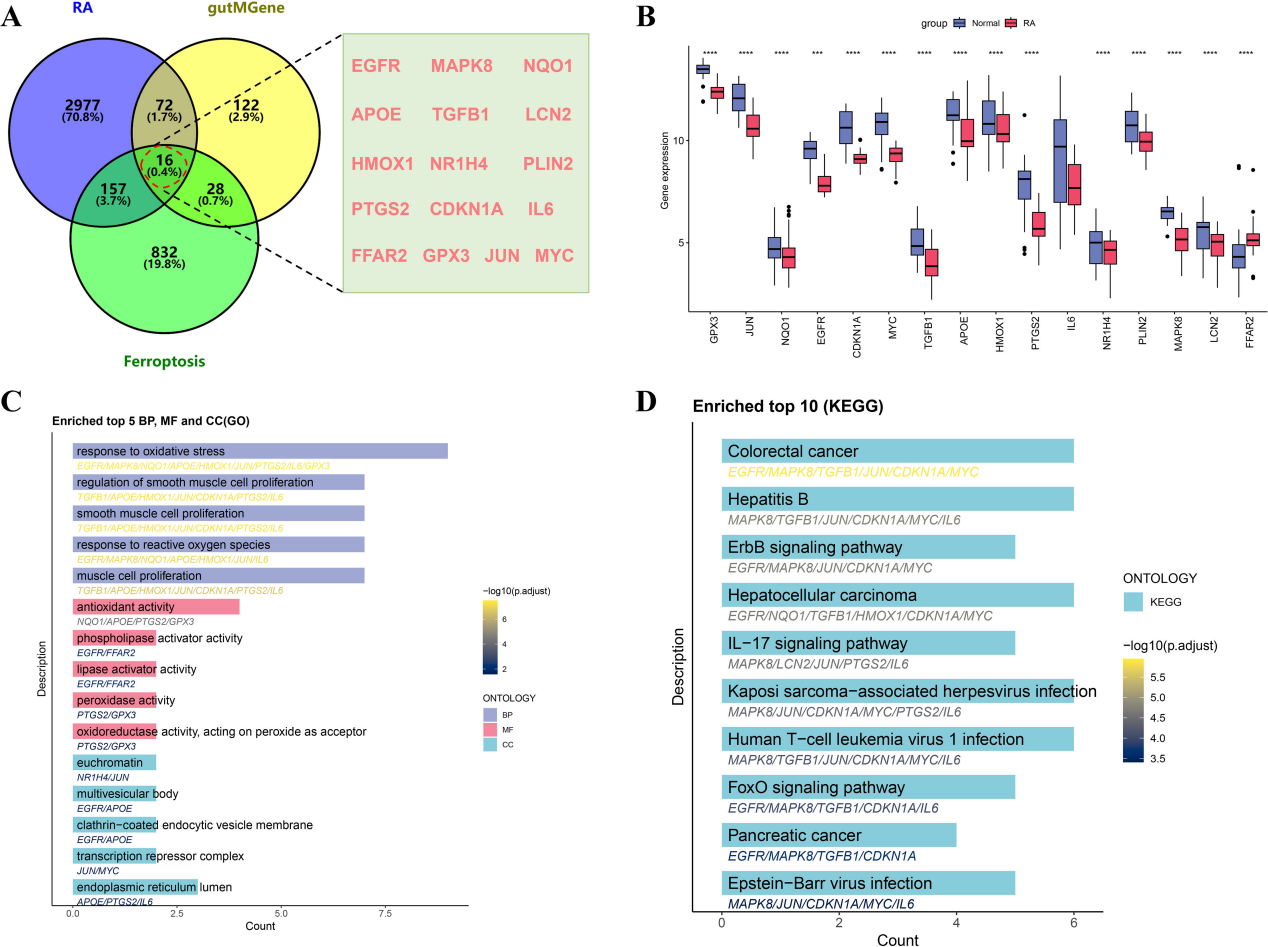
Screening and functional enrichment analysis of intersecting genes among RA, gutMGene, and ferroptosis. **(A)** Venn diagram showing the intersecting genes. **(B)** Boxplot comparing expression levels of intersecting genes between the RA and normal control groups. **(C)** Bar plot of GO enrichment analysis of intersecting genes, including BP, MF, and CC. **(D)** Bar plot of KEGG pathway enrichment analysis, showing the top 10 significantly enriched pathways of the intersecting genes. Significance: ****p < 0.0001.

By performing Venn diagram intersection analysis of gut microbiota targets, ferroptosis-related genes, and RA-associated genes, a total of 16 intersection genes were identified (**Figure 3A**). A bar plot of gene expression levels (**Figure 3B**) showed that all intersecting genes were significantly differentially expressed between RA and normal tissues, with antioxidant genes such as GPX3 and NQO1 significantly downregulated in RA tissues. To elucidate the biological functions and pathways associated with the intersecting genes, GO and KEGG enrichment analyses were performed. GO analysis revealed that these genes were mainly involved in oxidative stress response, regulation of cell proliferation, and lipid metabolism, with oxidative stress and reactive oxygen species responses potentially playing key roles in RA-related inflammation and ferroptosis (**Figure 3C**). In addition, these genes were enriched in molecular functions such as antioxidant enzyme activity, phospholipase regulation, and peroxidase activity, suggesting their crucial roles in immune regulation and metabolic processes in RA. KEGG enrichment analysis further revealed that these genes were broadly involved in inflammation- and immune-related pathways, including the IL-17 signaling pathway and FoxO signaling pathway (**Figure 3D**).

### 3.3 Machine learning-based identification of key genes

**Figure 4:**
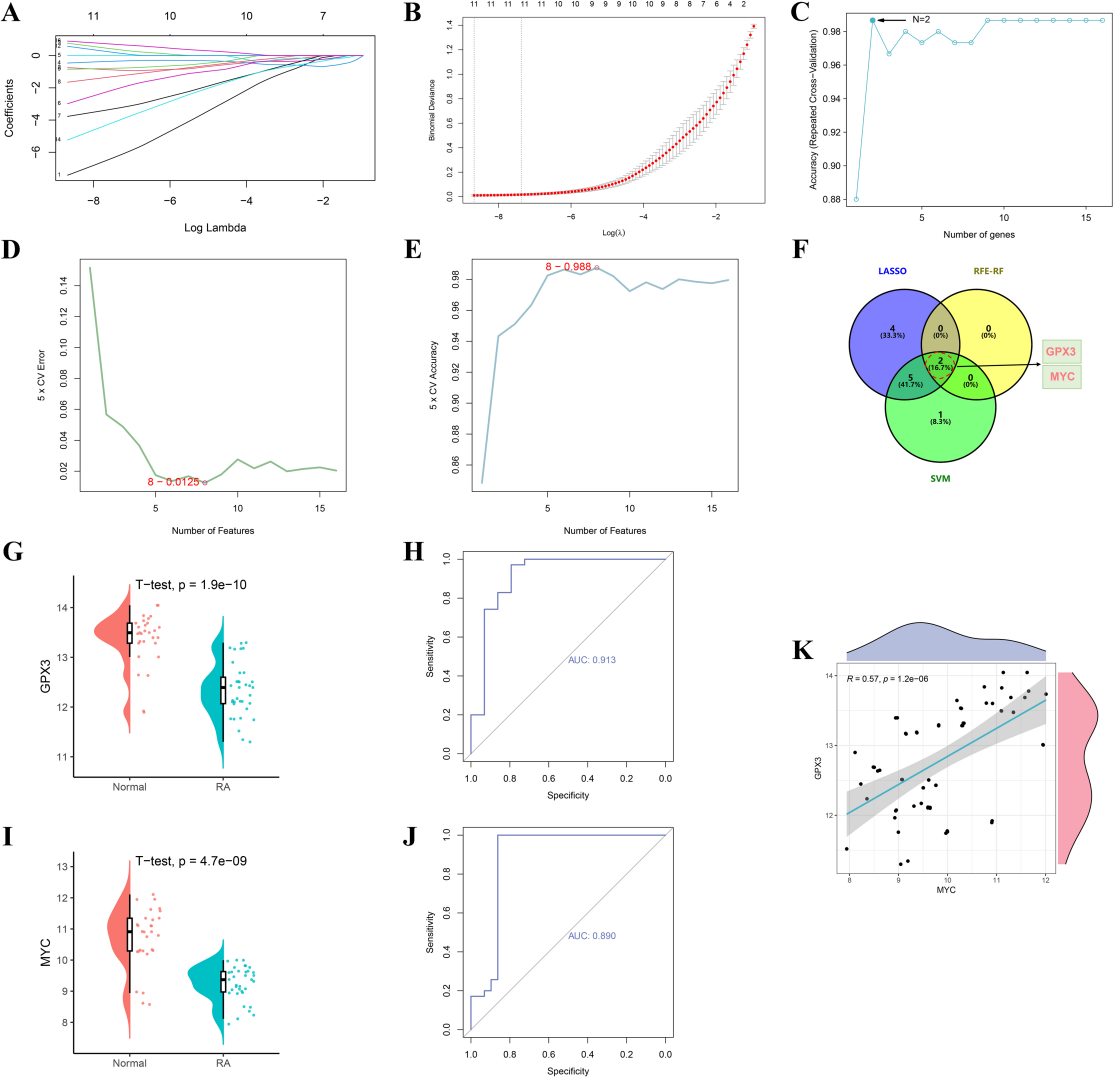
Screening and validation of RA–gut microbiota–ferroptosis key genes based on machine learning algorithms. **(A, B)** Feature selection of key genes using the LASSO regression algorithm. **(C)** Optimal number of feature genes determined by the RFE-RF algorithm. **(D, E)** Feature gene selection process using the SVM algorithm. **(F)** Venn diagram showing overlapping key genes identified by the three machine learning methods (LASSO, RFE-RF, and SVM). **(G–J)** Validation of GPX3 and MYC expression levels and ROC curve analyses. **(K)** Scatter plot showing the correlation between GPX3 and MYC expression levels.

To accurately identify core regulatory genes in RA, three machine learning algorithms were integrated to optimize analysis of the 16 intersecting genes. LASSO regression identified 11 key genes, and the coefficient shrinkage trajectory and cross-validation curve indicated good model stability (**Figures 4A, B**). RFE-RF analysis revealed that the model achieved the highest accuracy when two feature genes were selected, suggesting optimal performance in terms of gene selection accuracy and stability (**Figure 4C**). The SVM algorithm selected 8 genes and achieved a classification accuracy of 0.988 under this model (**Figures 4D, E**). By integrating results from the three machine learning methods, a Venn diagram was constructed to identify overlapping key genes, ultimately yielding GPX3 and MYC (**Figure 4F**). Expression analysis showed that GPX3 was significantly downregulated in RA tissues, with a single-gene ROC AUC of 0.913 (**Figures 4G, H**). MYC also showed significantly reduced expression, with an AUC of 0.890 (**Figures 4I, J**), indicating high sensitivity and specificity for RA diagnosis. Correlation analysis further revealed a significant positive correlation between GPX3 and MYC expression (R = 0.57, p = 1.2e-08), suggesting a potential synergistic role in RA pathogenesis (**Figure 4K**).

### 3.4 MLP model validation of key genes GPX3 and MYC

**Figure 5:**
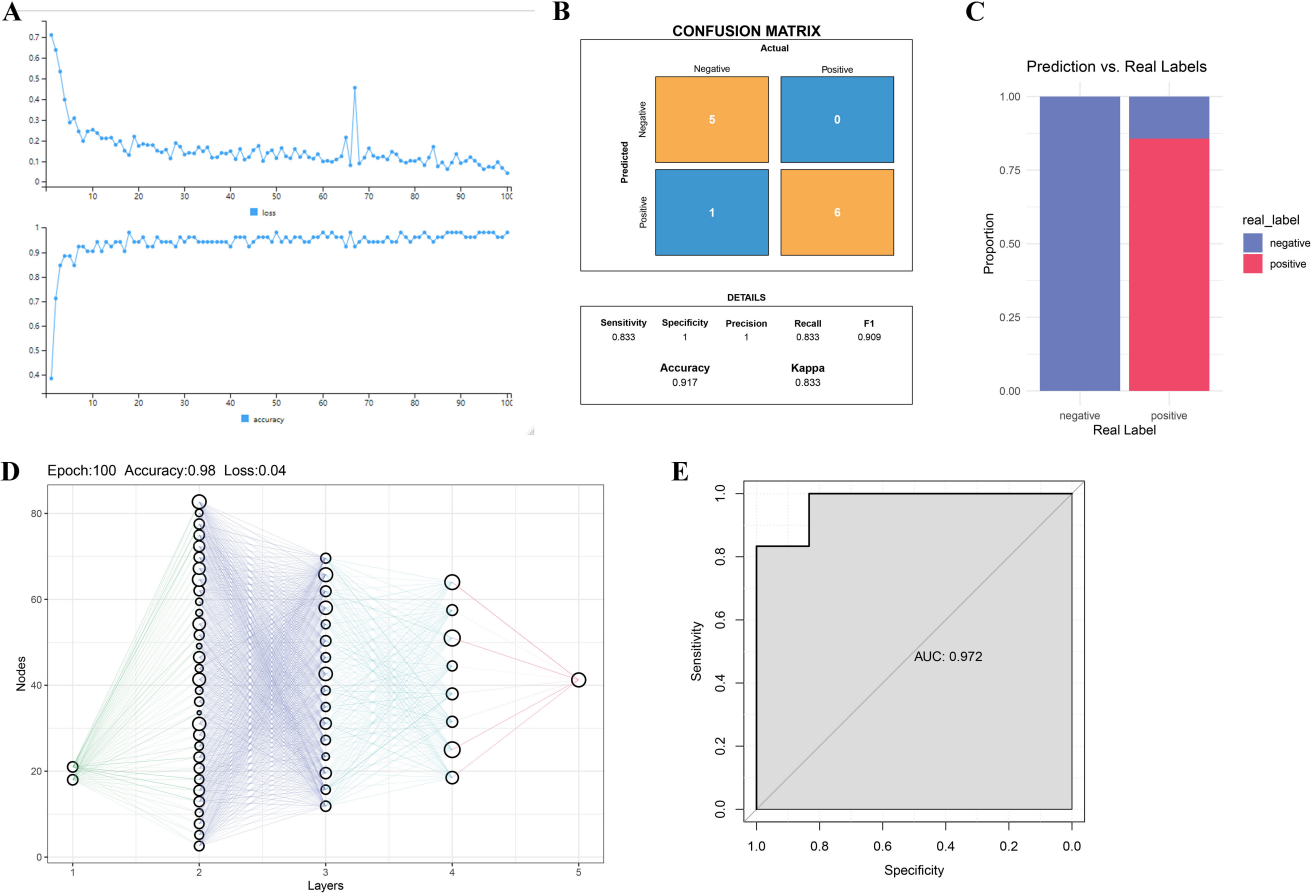
Validation of key gene-based RA diagnosis using the MLP model. **(A)** Loss and accuracy curves during MLP model training. **(B)** Confusion matrix of MLP model predictions. The x-axis represents actual classes, and the y-axis shows predicted classes. **(C)** Bar chart comparing real labels and model predictions. **(D)** Schematic of the MLP network structure, where nodes represent neurons and lines indicate inter-layer connections. **(E)** ROC curve analysis of the MLP model for RA classification.

To further validate the diagnostic value of GPX3 and MYC in RA, an MLP-based classification model was constructed and its predictive performance evaluated. **Figure 5A** shows the trends of loss and accuracy during MLP training. As training epochs increased, the loss steadily decreased and accuracy improved and stabilized, indicating good training performance without signs of overfitting or underfitting. The confusion matrix showed that the MLP model based on GPX3 and MYC achieved an accuracy of 0.917, sensitivity of 0.833, and recall of 0.833 on the test set, indicating strong classification performance between RA and normal groups (**Figure 5B**). **Figure 5C** further compares predicted outcomes with true labels, demonstrating that GPX3 and MYC can effectively distinguish between RA and normal tissues. The neural network visualization (**Figure 5D**) shows that GPX3 and MYC serve as input features, with classification completed through multiple hidden layers, highlighting their importance in RA diagnosis. The ROC curve of the model yielded an AUC of 0.972 (**Figure 5E**), indicating excellent discriminatory ability of the GPX3 and MYC-based model in RA prediction.

### 3.5 Single-gene GSEA and GSVA analysis of key genes

**Figure 6:**
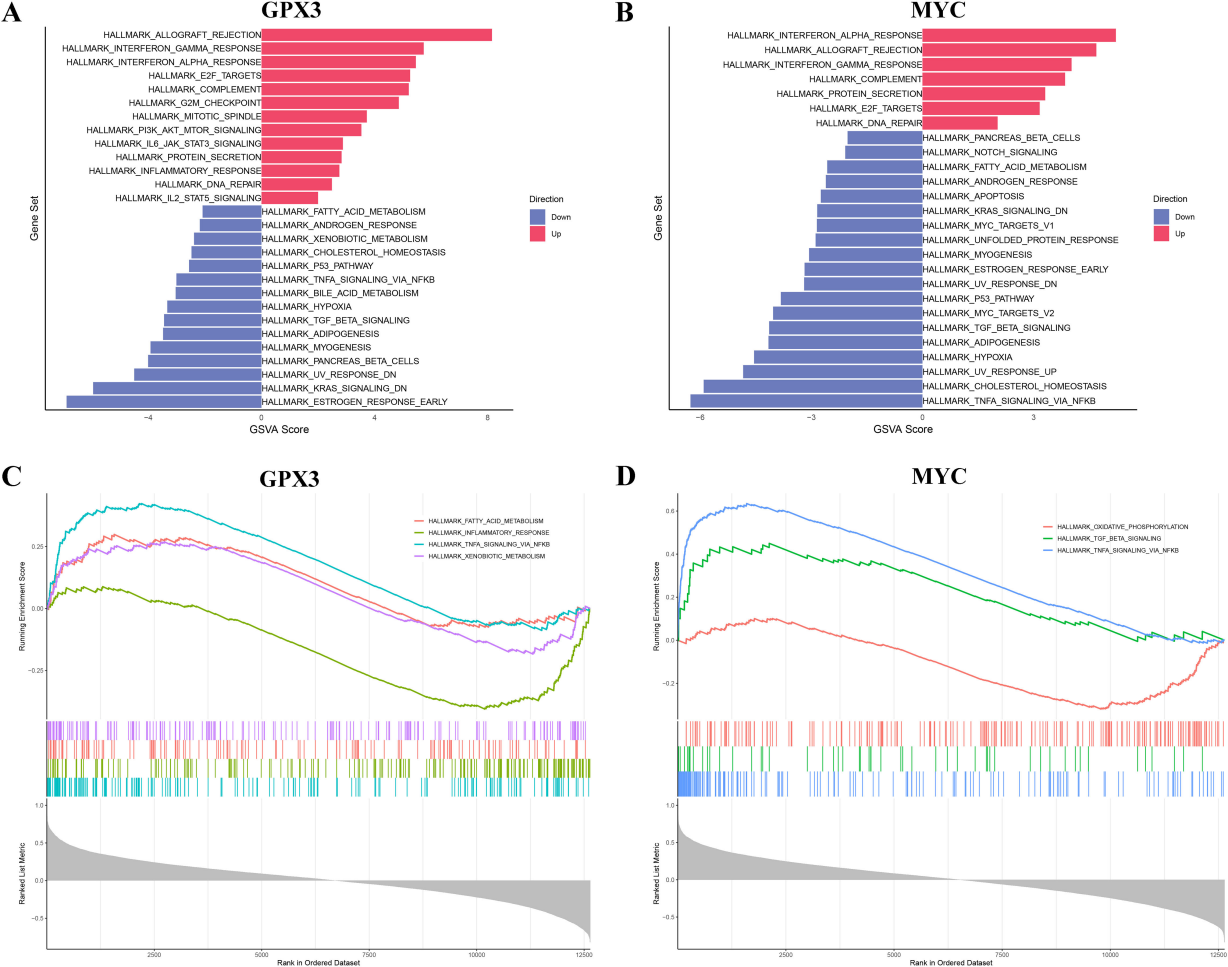
Functional enrichment analysis of key genes GPX3 and MYC via GSVA and GSEA. **(A, B)** GSVA results of GPX3 and MYC across different pathways. **(C, D)** GSEA enrichment plots for key genes GPX3 and MYC.

To further explore the potential functions of GPX3 and MYC in RA, GSVA and GSEA were employed to analyze their associated pathways. GSVA results showed that high expression of GPX3 and MYC was significantly associated with immune-related pathways, such as allograft rejection, interferon response, and inflammatory response, suggesting their involvement in remodeling the RA immune microenvironment (**Figures 6A, B**). Additionally, both genes showed negative correlations with lipid metabolism, cholesterol homeostasis, and the P53 pathway, suggesting they may influence RA progression by modulating metabolic homeostasis. GSEA analysis further confirmed the significance of GPX3- and MYC-associated pathways. GPX3 was mainly enriched in TNF-α mediated NF-κB signaling, steroid metabolism, and fatty acid metabolism pathways (**Figure 6C**), while MYC was primarily enriched in chromoprotein phosphorylation, TGF-β binding, and TNF-α mediated NF-κB signaling (**Figure 6D**). These findings indicate that GPX3 and MYC may play central roles in RA-related immune-inflammatory regulation and metabolic imbalance, potentially influenced by gut microbiota and their metabolites. In particular, lipid metabolism and NF-κB mediated inflammatory signaling, as key regulators of ferroptosis, further support the essential roles of GPX3 and MYC in the ferroptosis-related regulatory network in RA.

### 3.6 Immune infiltration analysis

**Figure 7:**
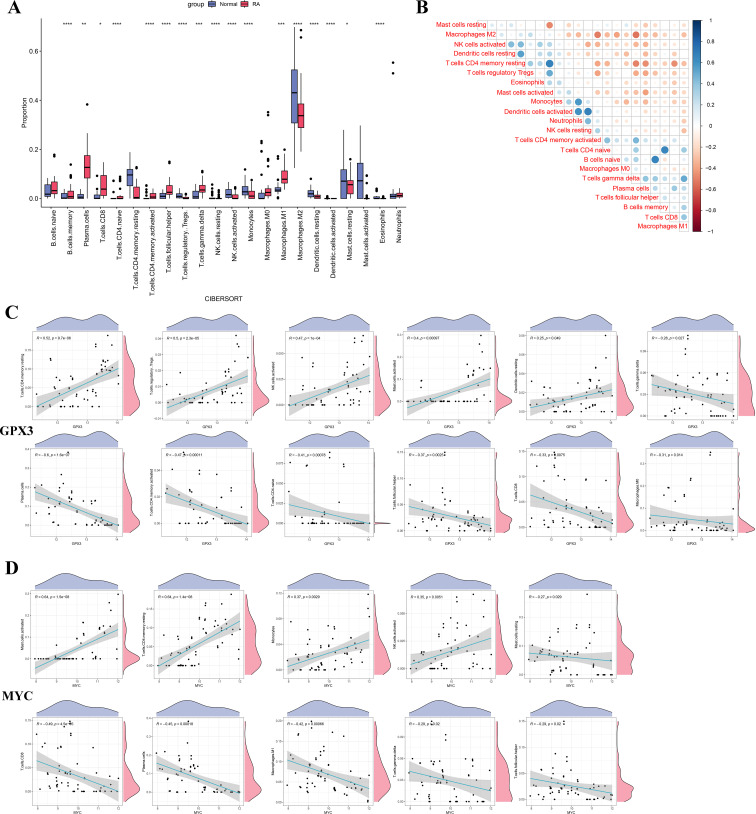
Differences in immune cell infiltration and correlation analysis between key genes and immune cells in RA. **(A)** Boxplot comparing the proportions of immune cell infiltration between the RA and normal control groups. **(B)** Heatmap showing the correlation analysis among immune cell types. **(C, D)** Correlation scatter plots between GPX3 and MYC expression levels and significantly altered immune cells. Significance: *p < 0.05; **p < 0.01; ***p < 0.001; ****p < 0.0001.

To investigate the potential roles of GPX3 and MYC in the RA immune microenvironment, CIBERSORT was used to estimate the infiltration of 22 immune cell types in RA and normal tissues (**Figure 7A**). The results showed that M1 macrophages and CD8+ T cells were significantly increased in RA tissues compared to normal tissues, while M2 macrophages, resting mast cells, and dendritic cells were decreased, indicating substantial alterations in the RA immune microenvironment characterized by increased pro-inflammatory and decreased anti-inflammatory cells, potentially contributing to persistent inflammation. **Figure 7B** shows correlations among immune cell types, revealing that M1 macrophages were positively correlated with CD8 T cells and negatively correlated with M2 macrophages, further supporting the presence of immune imbalance in RA. Additionally, we analyzed the correlations between GPX3 and MYC expression levels and the infiltration of different immune cell types (**Figures 7C, D**). GPX3 expression was significantly positively correlated with regulatory T cells (Tregs) and activated NK cells, and negatively correlated with CD8+ T cells and plasma cells. Similarly, MYC expression was positively correlated with activated NK cells and monocytes, and negatively correlated with CD8+ T cells and M1 macrophages. GPX3 and MYC may influence the RA immune microenvironment by regulating macrophage polarization, T cell function, and NK cell activation.

### 3.7 Single-cell expression and cell communication analysis of GPX3 and MYC

**Figure 8:**
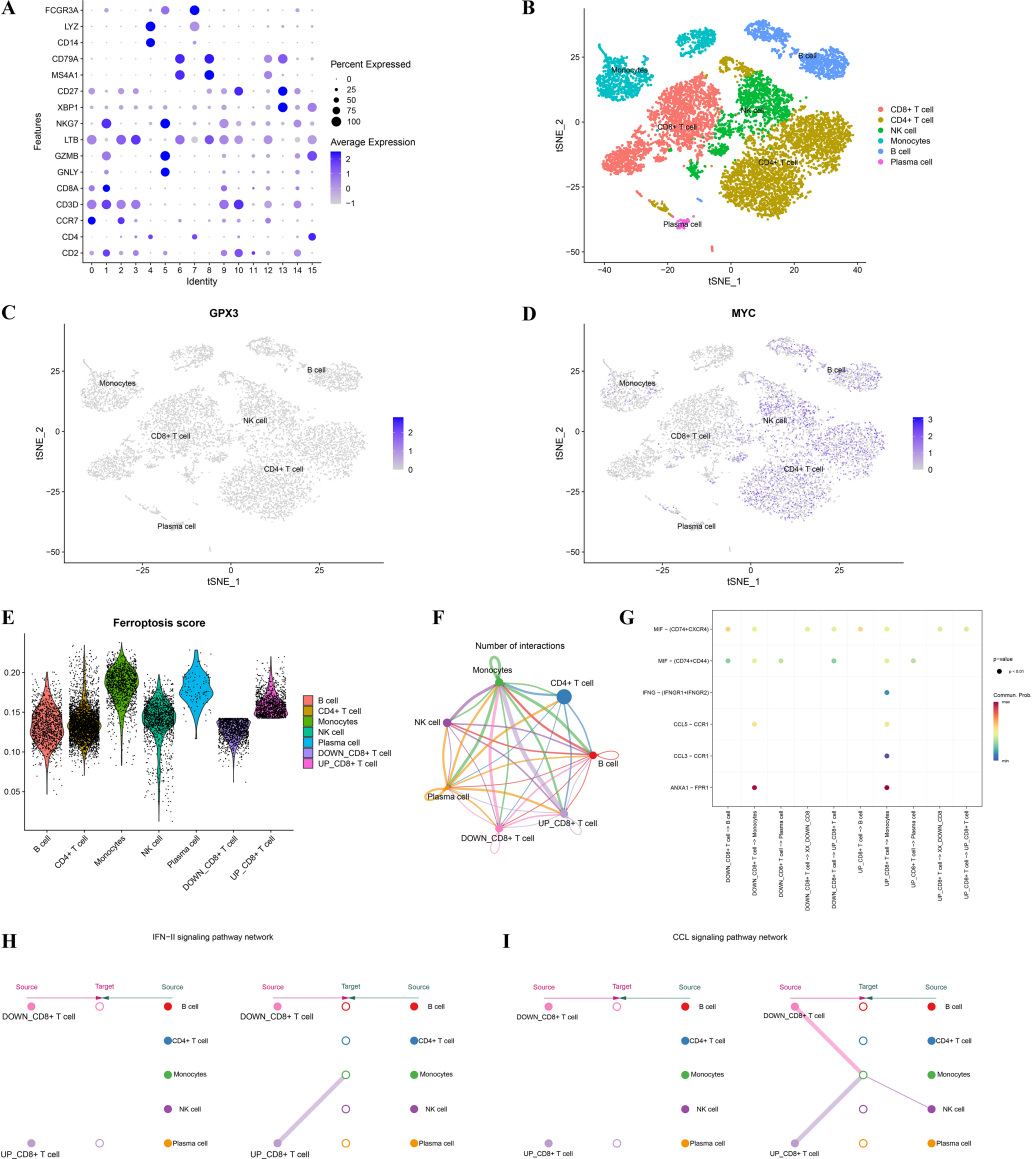
Single-cell analysis of GPX3 and MYC expression and their association with ferroptosis and the immune microenvironment. **(A)** Dot plot illustrating marker gene expression across different cell populations from single-cell data. **(B)** t-SNE dimensionality reduction clustering showing distribution and annotation of distinct immune cell clusters in RA single-cell data. **(C, D)** Expression patterns of key genes GPX3 and MYC across different cell clusters. **(E)** Violin plots showing differences in ferroptosis scores among immune cell populations. **(F)** Intercellular communication network, where edge colors and thickness represent the strength and frequency of cell-cell interactions. **(G)** Bubble plot displaying ligand–receptor interactions. **(H, I)** Network diagrams of IFN and CCL signaling pathways.

To further investigate the cell-specific expression patterns of GPX3 and MYC in the RA immune microenvironment and their roles in ferroptosis regulation, this study analyzed the cellular localization, ferroptosis scores, and cell communication patterns based on single-cell RNA sequencing (scRNA-seq) data. Based on known cell markers, we performed t-SNE dimensionality reduction clustering to classify RA peripheral blood mononuclear cells (PBMCs), identifying CD4^+^ T cells, CD8^+^ T cells, NK cells, monocytes, B cells, and plasma cells (**Figures 8A, B**). Further analysis of single-cell expression patterns of GPX3 and MYC (**Figures 8C, D**) showed GPX3 had very low expression restricted mainly to monocytes, while MYC was widely expressed in CD4^+^ T cells, NK cells, and B cells, suggesting MYC plays a critical role in T cell-mediated immune dysregulation in RA. Ferroptosis scores calculated using a ferroptosis-related gene set showed higher scores in monocytes and CD8^+^ T cells (**Figure 8E**). CD8^+^ T cells were further divided into high and low ferroptosis score groups, and cell communication analysis was performed using CellChat to construct interaction networks among RA immune cells (**Figure 8F**). Results showed extensive cellular communication between monocytes, CD8^+^ T cells, and B cells, indicating these cells may mediate RA immune regulation via ferroptosis-related signaling. Specific ligand-receptor analysis (**Figure 8G**) further revealed monocytes may influence T cell function through signaling axes such as CCL3-CCR1 and ANXA1-FPR1. Further analysis of key immune signaling pathways, such as the IFN-γ pathway (**Figure 8H**) and CCL signaling pathway (**Figure 8I**), revealed crucial cell-cell interactions involving CD8^+^ T cells, monocytes, and NK cells within the RA immune network.

### 3.8 GPX3–gut metabolite interactions: stability validation and dynamics characterization

**Figure 9:**
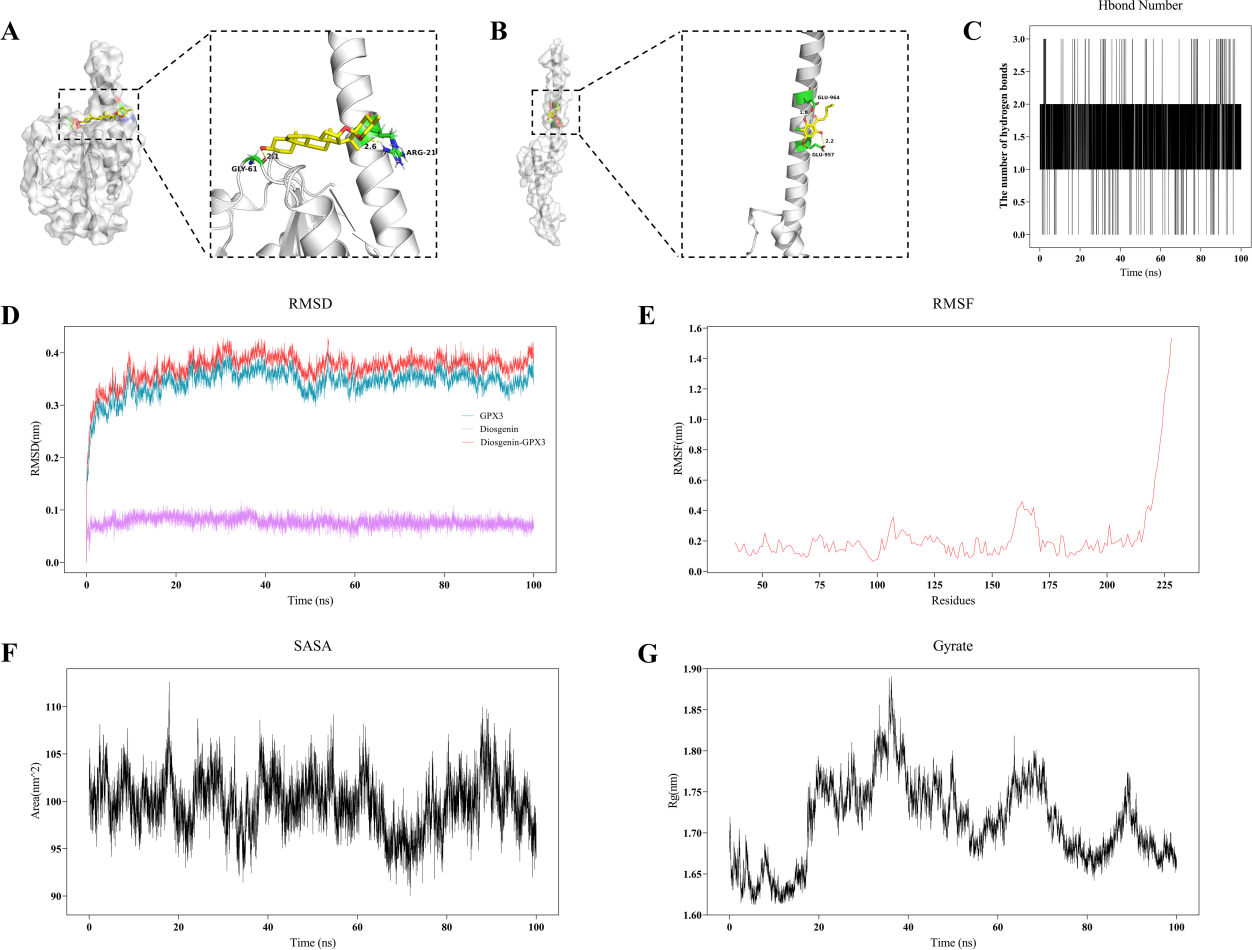
Molecular docking and molecular dynamics simulation analysis of gut microbiota metabolites and key genes. **(A, B)** 3D molecular docking visualization of gut microbiota metabolites with key proteins GPX3 **(A)** and MYC **(B)**. **(C)** Curve showing the changes in hydrogen bond numbers between metabolite and GPX3 during molecular dynamics simulations. **(D)** RMSD plot from molecular dynamics simulation of the GPX3–Diosgenin complex. **(E)** RMSF analysis plot of the GPX3–Diosgenin complex. **(F)** SASA change curve of the GPX3–Diosgenin complex during molecular dynamics simulation. **(G)** Gyration radius (Rg) plot of the GPX3–Diosgenin complex.

To investigate interactions between gut microbiota metabolites and key genes, molecular docking was performed initially, followed by molecular dynamics simulations to evaluate the binding stability and dynamic behavior of ligand–receptor complexes. **Supplementary Table 2** provides comprehensive details on critical binding residues, binding energies, and related parameters obtained during the molecular docking procedure. **Figure 9A** shows molecular docking results between GPX3 (2r37) and gut metabolite Diosgenin, indicating stable hydrogen bonds and hydrophobic interactions at the active site involving key residues GLY-61 and ARG-21, with a binding energy of -8.40 kcal/mol. Similarly, MYC (6g6k) interacted with gut metabolite Differentiation-inducing factor 3 (DIF-3), primarily via residues GLU-957 and GLU-964 (**Figure 9B**), with a binding energy of -5.41 kcal/mol. Based on this, a 100 ns molecular dynamics simulation of the GPX3-Diosgenin complex was conducted to further evaluate stability. Hydrogen bond analysis indicated the complex maintained a stable number of hydrogen bonds during simulation, suggesting strong binding of Diosgenin at GPX3’s active site (**Figure 9C**). RMSD trajectory analysis indicated that the overall structure of GPX3 stabilized upon Diosgenin binding, without significant conformational drift (**Figure 9D**). RMSF results showed that the core structure of GPX3 remained stable, with only minor fluctuations observed at the C-terminal region (**Figure 9E**). SASA analysis indicated that the solvent-accessible surface area of the GPX3–Diosgenin complex ranged from 95 to 110 nm², with no significant change in structural exposure after binding (**Figure 9F**). Rg analysis demonstrated that the complex maintained high structural compactness, remaining within the 1.65–1.85 nm range, further confirming that GPX3 retained a stable 3D conformation upon Diosgenin binding (**Figure 9G**). Additionally, the MMGBSA binding free energy of the GPX3-Diosgenin complex was -23.52 kcal/mol (**Supplementary Table 3**), indicating good conformational stability and reliable binding affinity of the complex. In summary, Diosgenin may modulate the function of GPX3 through stable binding, thereby influencing ferroptosis-related processes in RA.

## 4 Discussion

By integrating RA-related genes, gut microbiota-associated genes, and ferroptosis-related genes, this study systematically identified GPX3 and MYC as key genes using network pharmacology and various machine learning approaches. Further bioinformatics analyses revealed their potential mechanisms in RA, providing essential clues for understanding how gut microbial metabolites might influence RA progression via the ferroptosis pathway.

First, this study identified 3,222 RA-related genes through WGCNA and differential expression analysis. Intersection analysis using a Venn diagram of RA-related, gut microbiota-related, and ferroptosis-related genes identified, for the first time, 16 genes potentially mediating RA pathology and involved in gut microbiota metabolism and ferroptosis (EGFR, MAPK8, NQO1, APOE, TGFB1, LCN2, HMOX1, NR1H4, PLIN2, PTGS2, CDKN1A, IL6, FFAR2, GPX3, JUN, and MYC). Functional enrichment analysis indicated these intersecting genes are primarily involved in biological processes including oxidative stress responses, smooth muscle cell proliferation, and reactive oxygen species (ROS) responses, closely matching RA pathological features such as excessive inflammatory factor release and oxidative stress injury (16). Furthermore, these genes were mainly associated at the molecular function level with antioxidant activity, oxidoreductase activity, and lipase activity, further supporting their potential involvement in oxidative stress and inflammation regulatory pathways in RA pathogenesis. KEGG pathway analysis indicated these intersecting genes were significantly enriched in pathways including IL-17 signaling, FoxO signaling, and several viral infection-related pathways, with IL-17 and FoxO pathways playing critical roles in RA pathogenesis (17, 18). These findings suggest these genes may participate in RA pathogenesis through regulating immune-inflammatory responses and apoptosis-related pathways.

We further applied three classical machine learning algorithms (LASSO, RFE-RF, and SVM) to cross-validate and identify two core genes, GPX3 and MYC, which were subsequently validated using an MLP model. Both genes showed significant differential expression between RA patients and healthy controls, with ROC curve analysis demonstrating strong diagnostic performance (AUC values of 0.913 and 0.890, respectively). The three machine learning methods mentioned above have been widely used in feature selection and disease biomarker identification, each with its own strengths and limitations. LASSO provides strong variable selection capability, is suitable for handling high-dimensional data, and effectively reduces the risk of overfitting, though its results can be sensitive to the regularization parameter and performance may decrease in the presence of multicollinearity (19). RFE-RF combines the nonlinear modeling capability of random forests with a recursive feature selection mechanism, enabling it to capture complex relationships between features and the target variable, although stability may be limited when sample size is small (20). SVM, as a powerful classification tool particularly suitable for high-dimensional, small-sample data, demonstrates strong classification performance and generalization capability (21); however, it lacks interpretability and its feature selection depends heavily on kernel function selection and parameter tuning. Utilizing multiple algorithms for cross-validation can enhance the robustness of selection results from different perspectives, reduce bias risks associated with any single method, and increase the reliability of key gene identification. Results from this study suggest that GPX3 and MYC have potential diagnostic value in RA.

GPX3 encodes glutathione peroxidase 3, an important antioxidant enzyme primarily responsible for reducing oxidative damage by catalyzing the conversion of hydrogen peroxide into water (22). Although few studies have examined the relationship between GPX3 and RA, existing evidence suggests GPX3 has significant antioxidant activity and may protect synovial tissues from oxidative stress damage in RA (23). Additionally, GPX3 can resist autophagy-related ferroptosis via the AMPK/mTOR signaling pathway (24). Synovial cells in RA patients remain chronically inflamed, exhibiting significantly elevated iron metabolism disorder and oxidative stress levels, potentially exacerbating joint damage and inflammatory cell infiltration (25). Thus, decreased GPX3 expression might impair tissue antioxidative capacity, exacerbating pathological damage (26). Future studies should investigate GPX3’s specific impact on oxidative stress-related RA pathology in greater detail. MYC, a critical transcription factor, plays an essential role in biological processes such as cell proliferation, differentiation, and apoptosis (27). Studies have demonstrated that MYC participates in various pathological aspects of RA, including synovial cell proliferation, osteoclast activation, immune inflammation, and cartilage damage. Specifically, elevated MYC expression in RA synovial fibroblasts significantly enhances abnormal cell proliferation, migration, and invasion, worsening synovial inflammation and joint destruction (28, 29). Additionally, MYC can enhance osteoclast differentiation and bone-resorption activity by regulating transcription of glutamine transporter (Slc1a5) and glutaminase (Gls1), accelerating bone erosion in RA patients (30, 31). Furthermore, MYC mediates macrophage metabolic reprogramming, elevating glycolysis levels and promoting M1 pro-inflammatory macrophage polarization and inflammatory cytokine expression, thus exacerbating synovial inflammation in RA (32).

This study further elucidated the potential mechanisms of GPX3 and MYC in RA through GSVA and GSEA analyses. High expression of GPX3 and MYC was significantly enriched in interferon-α/γ responses and TNF-α/NF-κB signaling pathways, whereas low expression was enriched in metabolic pathways such as fatty acid metabolism, bile acid metabolism, and oxidative phosphorylation. These pathways have been widely reported to play crucial roles in RA inflammation and metabolic disorders (33–35). Previous studies indicated that persistent activation of interferon and TNF-α signaling directly results in sustained activation of inflammatory cells in RA synovial tissues, exacerbating inflammation and tissue damage (36, 37). Meanwhile, lipid metabolism abnormalities and mitochondrial oxidative phosphorylation dysfunction may trigger ferroptosis, exacerbating RA synovial tissue damage through the accumulation of lipid peroxides (38). These results suggest that GPX3 and MYC might regulate ferroptosis in RA through the aforementioned inflammatory and metabolic pathways, thereby exacerbating disease progression. To further explore the specific roles of GPX3 and MYC in the immune microenvironment, we performed single-cell sequencing-based ferroptosis scoring of different immune cell populations in RA patients, particularly focusing on communication differences between high and low ferroptosis-scoring CD8+ T-cell subpopulations and other immune cells. Analysis revealed significantly enhanced IFN-II and CCL signaling interactions between high ferroptosis-scoring CD8+ T cells and monocytes, NK cells, and CD4+ T cells, while CD8+ T cells with low ferroptosis scores exhibited weaker intercellular signaling. These findings align with the GSEA analysis results mentioned above, where enhanced interferon signaling pathways significantly promoted inflammatory cell infiltration and synovial tissue damage (36, 39, 40). Additionally, CCL3 signaling is closely associated with inflammatory infiltration and immune cell recruitment in RA synovial tissues (41, 42). Therefore, the cell communication results clearly support the hypothesis that CD8+ T cells with high ferroptosis levels enhance interactions with other immune cells through these inflammatory signaling pathways, exacerbating synovial inflammation and injury.

Finally, we explored the potential interaction mechanisms between gut microbial metabolites and GPX3 protein through molecular docking and molecular dynamics simulation analyses. Molecular docking indicated that the gut metabolite Diosgenin binds tightly to GPX3, forming multiple stable hydrogen bonds at residues GLY-61 and ARG-21. DIF-3 also showed strong binding affinity to MYC. This suggests that gut metabolites may directly modulate the function or expression of GPX3 and MYC, thereby affecting the pathological processes of RA. Subsequently, molecular dynamics simulations of the GPX3-Diosgenin complex were conducted, with RMSD, RMSF, SASA, and Gyrate analyses revealing a compact and stable conformation.

Existing studies indicate that gut microbiota metabolites participate in regulating host ferroptosis through various mechanisms, such as modulating glutathione metabolism, reactive oxygen species (ROS) clearance, and lipid peroxidation (43). Combining the previously discussed analyses of GPX3 and MYC with GSVA and GSEA pathway enrichment results, the downregulation of GPX3 can lead to ROS accumulation, inducing lipid peroxidation and subsequently triggering ferroptosis in synovial cells. Conversely, MYC upregulation may enhance glycolytic activity in macrophages, promoting their polarization toward the M1 phenotype, thereby creating a vicious cycle of inflammation, metabolism, and cell death, exacerbating local immune imbalance and tissue destruction in RA. Gut-derived metabolites such as Diosgenin and DIF-3 demonstrated strong binding stability in molecular docking and molecular dynamics simulations, suggesting their potential role in regulating GPX3 and MYC functions, thus modulating oxidative stress and ferroptosis processes in synovial cells. Based on the above assumptions, we propose a novel potential intervention strategy: targeting GPX3 and MYC by modulating specific gut microbiota metabolites (such as Diosgenin and DIF-3), thus achieving multi-level regulation of the RA pathological process. On one hand, developing small-molecule modulators targeting GPX3/MYC could enhance antioxidant defense capabilities or intervene in immunometabolic reprogramming; on the other hand, approaches such as oral probiotic administration or dietary polyphenol intake could modulate gut microbiota composition, optimizing its metabolite profile. In the future, it will be necessary to systematically validate the “gut microbiota–metabolite–ferroptosis” axis by combining animal experiments with integrated metagenomic and metabolomic analyses, providing theoretical foundations and practical pathways for precision treatment of RA.

In conclusion, through comprehensive bioinformatics analysis and multiple machine learning methods, this study successfully identified and validated GPX3 and MYC as two key genes related to gut microbial metabolism and ferroptosis in RA progression. Both key genes showed significant differential expression in RA patients, were closely associated with immune cell infiltration and inflammatory signaling pathways, and exhibited high clinical value for disease diagnosis. Furthermore, this study elucidated the specific mechanisms by which GPX3 and MYC may contribute to RA from the perspectives of gut microbial metabolism and ferroptosis, providing valuable clues for further exploring RA pathogenesis and discovering novel therapeutic targets.

## Data Availability

Publicly available datasets were analyzed in this study. This data can be found here: GSE12021 (https://www.ncbi.nlm.nih.gov/geo/query/acc.cgi?acc=GSE12021); GSE55235 (https://www.ncbi.nlm.nih.gov/geo/query/acc.cgi?acc=GSE55235); GSE55457 (https://www.ncbi.nlm.nih.gov/geo/query/acc.cgi?acc=GSE55457); GSE159117 (https://www.ncbi.nlm.nih.gov/geo/query/acc.cgi?acc=GSE159117).
