# Supplementary material for: Integrative multi-omics analysis reveals the interaction mechanisms between gut microbiota metabolites and ferroptosis in rheumatoid arthritis
Source: Front Immunol. 2025 Jul 9;16:1608262. doi: 10.3389/fimmu.2025.1608262 (PMC12283288; doi:10.3389/fimmu.2025.1608262)
Supplement: Supplementary file 4 [file Table4.docx]

Supplementary Material


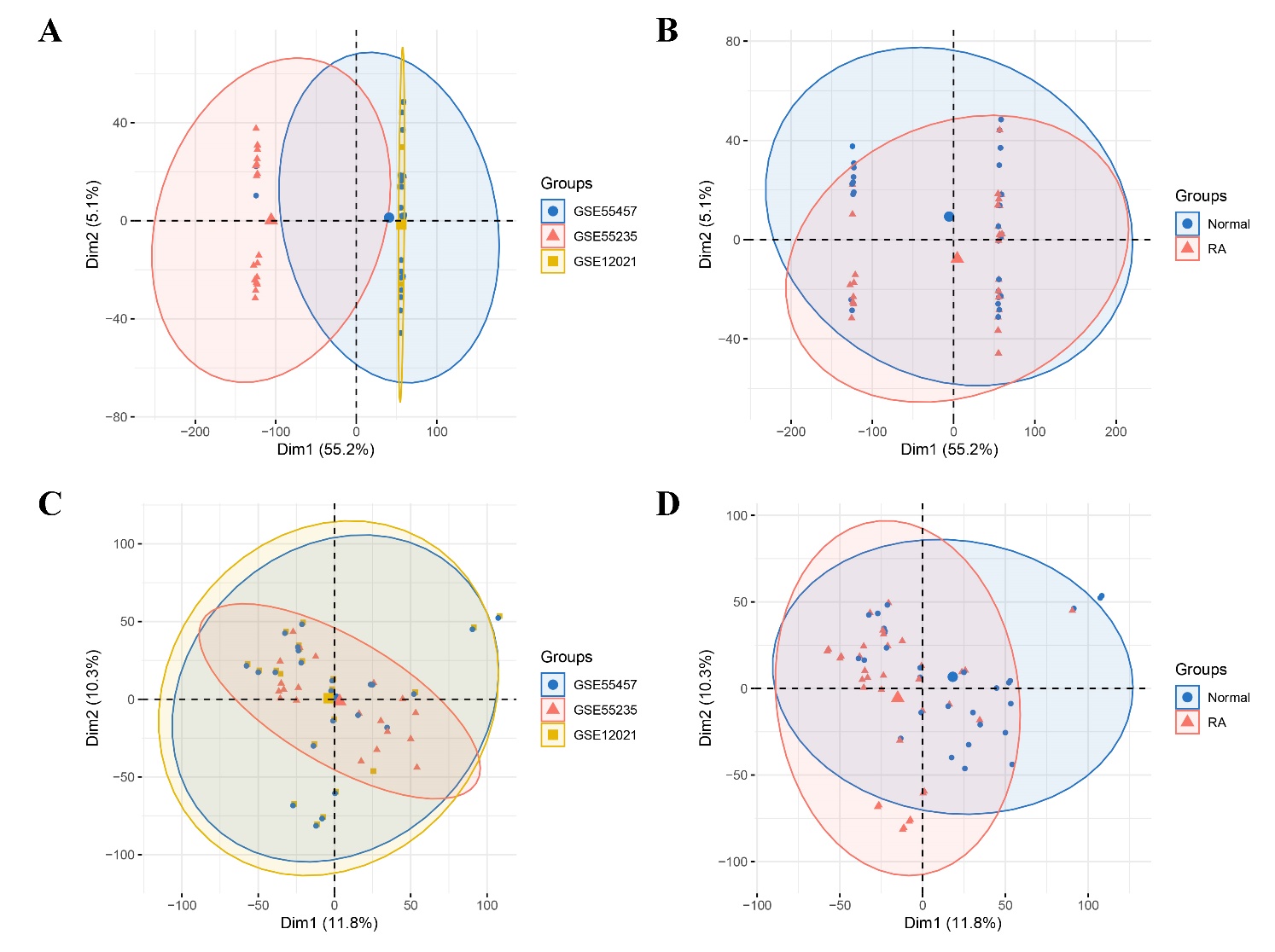


**Supplementary Figure 1**

PCA visualization analysis of dataset integration and batch effect correction. **(A–B)** show the PCA results of samples before batch effect correction, **(A)** grouped by dataset origin; **(B)** grouped by disease status. **(C–D)** show the PCA results after batch effect correction.


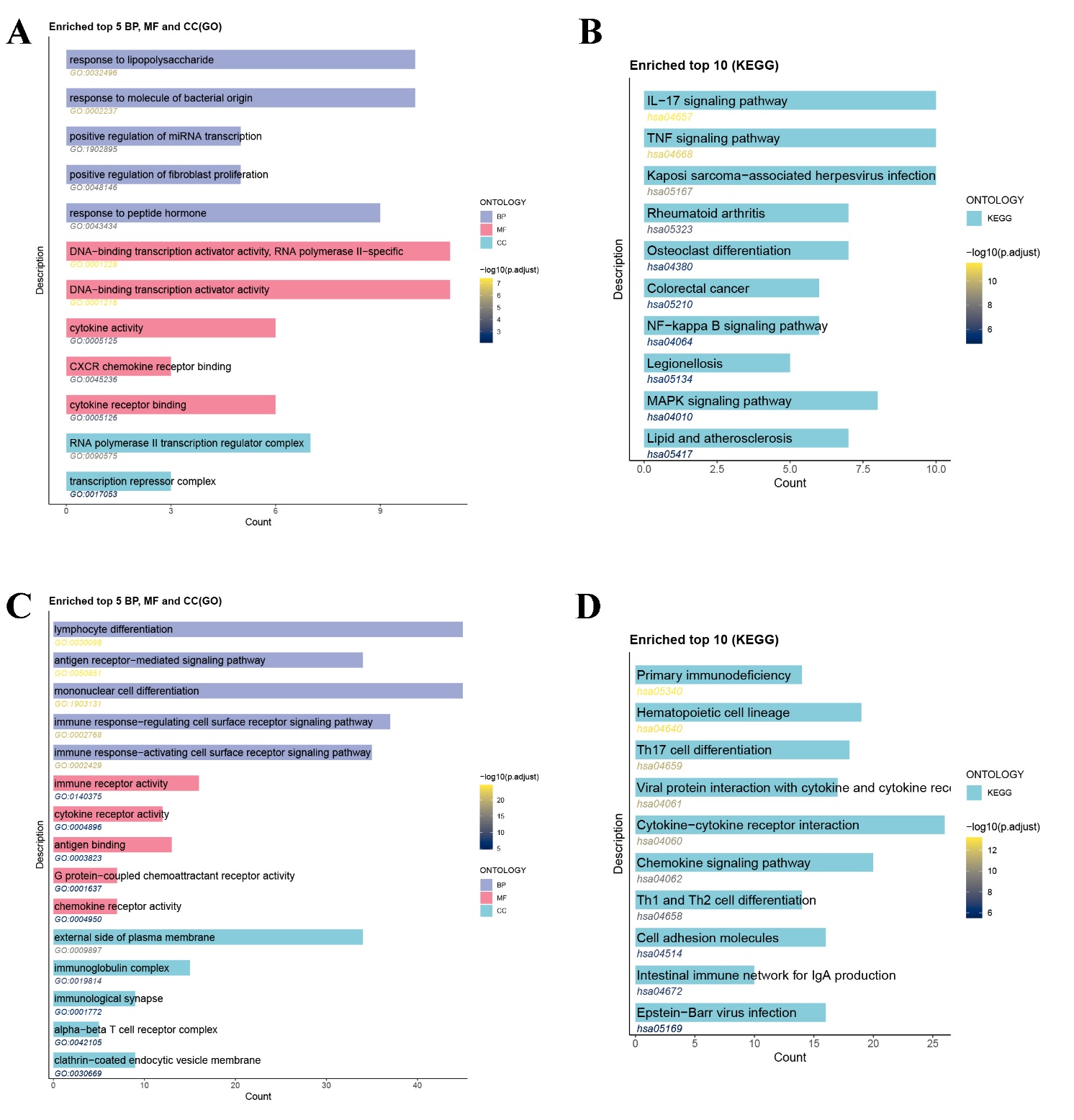


**Supplementary Figure 2**

GO and KEGG enrichment analysis results of the blue and turquoise modules. **(A–B)** show the enrichment analysis results for genes in the blue module, where **(A)** displays significantly enriched GO terms (including BP, MF, and CC), and **(B)** shows significantly enriched KEGG pathways; **(C, D)** show the enrichment analysis results for genes in the turquoise module, where **(C)** displays GO terms, and **(D)** displays KEGG pathways.
